# In search of the ideal risk-scoring system for very high-risk cardiac surgical patients: a two-stage approach

**DOI:** 10.1186/s13019-016-0405-3

**Published:** 2016-01-19

**Authors:** Marco Ranucci, Umberto Di Dedda, Serenella Castelvecchio, Maria Teresa La Rovere, Lorenzo Menicanti, Surgical and Clinical Outcome Research (SCORE) Group

**Affiliations:** Department of Cardiothoracic and Vascular Anesthesia & ICU, IRCCS Policlinico San Donato, Via Morandi 30, 20097, San Donato Milanese, Milan, Italy; Department of Cardiac Surgery, IRCCS Policlinico San Donato, Milan, Italy; Departments of Cardiology, and Biomedical Engineering, Fondazione Salvatore Maugeri, IRCCS Istituto Scientifico di Montescano, Montescano, Italy

**Keywords:** Risk stratification, Cardiac surgery, Anemia, Pulmonary hypertension

## Abstract

**Background:**

Cardiac surgery patients at very high risk are difficult to stratify with the existing risk scores. The objective of this study is to assess the clinical performance of two existing risk stratification scores (EuroSCORE II and ACEF score) in the setting of very high-risk patients undergoing cardiac surgery, and to identify a possible strategy to better address this patient population.

**Methods:**

Three-thousand-four-hundred-twenty eight (3,428) consecutive adult patients receiving cardiac operations at a single institution were investigated. Patients having an operative mortality risk >25 % at either the EuroSCORE II or the ACEF score were selected (105 patients). The discrimination power and calibration of the EuroSCORE II and the ACEF score were investigated. Factors associated with operative mortality were included in a multivariable logistic regression model and a new model was re-built for this patient population.

**Results:**

The observed mortality rate was 26 %. The expected mortality rate was underestimated by the EuroSCORE II (16 %) and overestimated by the ACEF Score (36 %). The EuroSCORE maintained a good discrimination (c-statistics 0.75) while the ACEF score did not (c-statistics 0.52). Within this patient population, the independent risk factors for operative mortality were emergency surgery, serum creatinine levels, pulmonary hypertension, and preoperative anemia. A model based on these factors provided an expected mortality risk of 26 % with a good discrimination (c-statics 0.82). Applying this model to extremely high-risk patients (expected mortality rate > 50 %) resulted in the re-classification of 25 % of the patient population.

**Conclusions:**

The existing risk models have a poor clinical relevance in the segment of patients at very high mortality risk. This is particularly frustrating, because these patients are those where the decision-making process is more important. A two-stage classification strategy (first stage: EuroSCORE II/ACEF score risk > 25 %; second stage: reclassification based on pulmonary hypertension, serum creatinine, and anemia) seems a possible strategy to correctly address very high-risk patients.

## Background

Risk stratification in cardiac surgery patients is usually performed using the three risk stratification scores recently accepted by the joint guidelines of the European Association for Cardio-Thoracic Surgery (EACTS) and the European Society of Cardiology (ESC) [1]. These are the European System for Cardiac Operative Risk Evaluation version II (EuroSCORE II) [2], the STS-PROM Score [3], and the age, creatinine, ejection fraction (ACEF) score [4].

The recently released EuroSCORE II is probably the most widely used mortality risk scoring system in Europe. However, a number of studies have underlined its underestimation of the mortality risk in high-risk cardiac surgery patients. The first validation study, published a few months after the publication of the EuroSCORE II, confirmed the good discriminative power of this tool (c-statistics 0.81), but noticed that in very high-risk patients the observed mortality rate (11 %) was higher than the expected (6.5 %) [5]. Subsequently, other studies confirmed similar findings: in combined cardiac surgery, the observed mortality rate was almost double the expected [6]; in urgent and emergent procedures the observed mortality rate exceeded the expected by 43 % and 45 % respectively [7], and by 25 % in high-risk (expected mortality rate > 9.2 %) patients [7]. In another series, the observed mortality (28 %) was double the expected (14 %) in patients with an expected mortality > 10 % [8]. The underestimation of mortality in high-risk patients was confirmed in a large UK series [9] and by a recent meta-analysis [10].

Losing reliability of mortality risk prediction in high- or very-high risk patients is frustrating for cardiac surgeons. The segment of patients with an expected mortality rate >20 %–25 % represents a challenge for the therapeutic decision-making process. Within this patient population, different options are often viable: conventional surgery; alternative procedures like transcatheter aortic valve replacement or mitral valve repair; aortic valve balloon angioplasty; percutaneous transluminal coronary angioplasty; and pharmacological therapy. Therefore, there is an unmet need for an accurate surgical mortality risk prediction.

The aims of the present study are (i) to verify if the existing risk scores are actually unreliable within the segment of patient population at very high surgical mortality risk, and (ii) to search additional factors which may improve the mortality risk prediction in this patient population.

## Methods

### Study design

This is a retrospective cohort study based on our institutional database for cardiac surgery patients. The period considered was October 2011 to January 2015. In this period, all the patients received a preoperative risk assessment based on the EuroSCORE II, plus the usual ACEF Score assessment already in place at our institution since 2010. The STS-PROM is not routinely utilized at our institution. The local ethics committee (IRRCS San Raffaele Hospital) approved the experimental design and waived the need for a written informed consent from the patients, who however gave a written consent to the scientific treatment of their data for scientific purposes and in anonymous form.

### Patients

During the time period considered 4,756 patient were operated. Patients receiving surgery for congenital heart disease were excluded (1,328 subjects). The remaining 3,428 patients were analyzed for operative mortality risk, and the patients with an expected mortality risk > 25 % at either the EuroSCORE II or the ACEF score were extracted and constituted the study group (105 subjects). This was considered the first-stage risk assessment.

We did not include an assessment of the third accepted risk stratification system (STS-PROM) in this patient population. This is due to the fact that the STS-PROM supports only a limited number of procedures (isolated coronary surgery, isolated mitral/aortic valve surgery, and combined coronary surgery + mitral/aortic valve surgery), and in our series, 35 patients (33 %) did not belong to these categories.

### Data collection and definitions

All data were retrieved from our institutional database. The EuroSCORE II was calculated for every single patient enrolled in the series with the EuroSCORE II interactive calculator, available online (www.euroscore.org). For each patient the mortality risk was assessed with the ACEF score too.

Preoperative data routinely collected in our institutional database include all the risk factors considered in the EuroSCORE II, plus a number of additional pre- and intraoperative data. All the definitions are in agreement with those reported in the original EuroSCORE II presentation [2]. The postoperative outcome data included low cardiac output state (inotropic drugs > 48 h), use of intra-aortic balloon pump (IABP) or ventricular assistance, acute kidney injury (AKI, defined as peak postoperative serum creatinine level > double the baseline), stroke, surgical revision, mesenteric infarction, systemic infections, and operative mortality (in-hospital or within 30 days from surgery for patients discharged).

The patient population was divided into two groups (survivors and non-survivors) according to the presence of operative mortality.

### Statistics

The EuroSCORE II and the ACEF score were assessed for discrimination power and calibration in the study population. Discrimination of each model was assessed with Receiver Operating Characteristics (ROC) analyses considering the area under the curve (AUC) and its 95 % confidence intervals. The calibration was assessed with the Hosmer-Lemeshow test, and as a further measure of calibration and clinical performance the expected mortality rate was compared to the observed mortality rate in terms of percentage with 95 % confidence interval.

The two groups (survivors and non-survivors) were analyzed for differences in preoperative variables. Differences between frequencies were analyzed with a Pearson’s chi-square, differences between continuous variables were analyzed with a two-sided Student’s *t* test (for normally distributed variables) and non-parametric tests (for non-normally distributed variables).

Factors being associated with operative mortality at the univariate analysis were entered into a multivariable logistic regression analysis (stepwise forward) to check their role as independent predictors of mortality. This was expressed in terms of odds ratios with 95 % confidence interval. To avoid overfitting of the model, a maximum of 1 factor per 10 mortality events was allowed. Multicollinearity was checked.

This second-stage model of risk stratification was assessed in terms of clinical performance using a ROC analysis and re-classification tables.

All data were expressed as number and percentage with 95 % confidence interval, mean and standard deviation (SD) of the mean or median and interquartile range when appropriate. All the tests are two-sided, and a *P* value < 0.05 was considered significant. Statistical analysis was performed using a computerized package SPSS 13.0 (SPSS Inc, Chicago, IL).

## Results

Out of the 105 patients who were defined at high operative risk (>25 %) according to the EUROSCORE II (*n* = 21); to the ACEF score (*n* = 78), or both (6), 27 (26 %) patients had the end-point of operative mortality.

The preoperative profile and operative data of the total patient population and of survivors/non survivors are shown in Table 1. At an univariate analysis, the patients who did not survive had a higher rate of pulmonary hypertension (PH, systolic pulmonary artery pressure > 55 mmHg), a higher serum creatinine value, a lower preoperative hematocrit (HCT) and were more likely to be operated under emergency conditions and to receive isolated valve surgery.

**Table 1:** Preoperative conditions and type of surgery in the patient population

Factor	Total population	Survivors	Non-survivors	*P*
	*N* = 105	*N* = 78	*N* = 27	
Age (years)	74.5 (7.2)	74.9 (6.8)	73.5 (8.3)	0.404
Weight (kgs)	71.2 (12.9)	70.4 (11.6)	73.5 (16.2)	0.290
Gender female	27 (25.7)	18 (23.1)	9 (33.3)	0.293
Left ventricle EF (%)	26 (23–30)	25 (23–30)	26 (23–33)	0.514
Pulmonary hypertension	13 (12.4)	6 (7.7)	7 (25.9)	0.013
Recent (30 days) MI	7 (6.7)	6 (7.7)	1 (3.7)	0.474
Unstable angina	1 (1.0)	1 (1.3)	0 (0)	0.554
Congestive heart failure	29 (27.6)	20 (25.6)	9 (33.3)	0.441
Cardiogenic shock	6 (5.7)	3 (3.8)	3 (11.1)	0.161
Intra-aortic balloon pump	7 (6.7)	4 (5.1)	3 (11.1)	0.283
Active endocarditis	5 (4.8)	3 (3.8)	2 (7.4)	0.454
COPD	18 (17.1)	15 (19.2)	3 (11.1)	0.335
Previous CVA	10 (9.5)	8 (10.3)	2 (7.4)	0.664
Diabetes on medication	31 (29.5)	22 (28.2)	9 (33.3)	0.615
Serum creatinine (mg/dL)	1.4 (1.2–1.8)	1.3 (1.2–1.6)	1.7 (1.4–1.9)	0.002
Serum bilirubin (mg/dL)	0.5 (0.5–0.7)	0.5 (0.5–0.7)	0.5 (0.5–0.6)	0.957
Hematocrit (%)	35.8 (5.5)	36.5 (5.3)	34.0 (5.8)	0.047
Redo surgery	17 (16.2)	10 (12.8)	7 (25.9)	0.111
Urgent procedure	37 (35.2)	28 (35.9)	9 (33.3)	0.810
Emergent procedure	7 (6.7)	2 (2.6)	5 (18.5)	0.004
Isolated CABG	25 (23.8)	22 (28.2)	3 (11.1)	0.074
CABG + mitral valve	19 (18.1)	16 (20.5)	3 (11.1)	0.274
CABG + aortic valve	8 (7.6)	5 (6.4)	3 (11.1)	0.427
Mitral + aortic valve	5 (4.8)	3 (3.8)	2 (7.4)	0.454
Ascending aorta/arch	5 (4.8)	3 (3.8)	2 (7.4)	0.454
Isolated valve	23 (21.9)	13 (16.7)	10 (37.0)	0.027
Others	20 (19)	16 (20.5)	4 (14.8)	0.516

Data are number (%) or mean (standard deviation) or median (interquartile range)

*CABG* coronary artery bypass graft, *CVA* cerebrovascular accident, *COPD* chronic obstructive pulmonary disease, *EF* ejection fraction

### Observed operative mortality and risk stratification

**Fig. 1:**
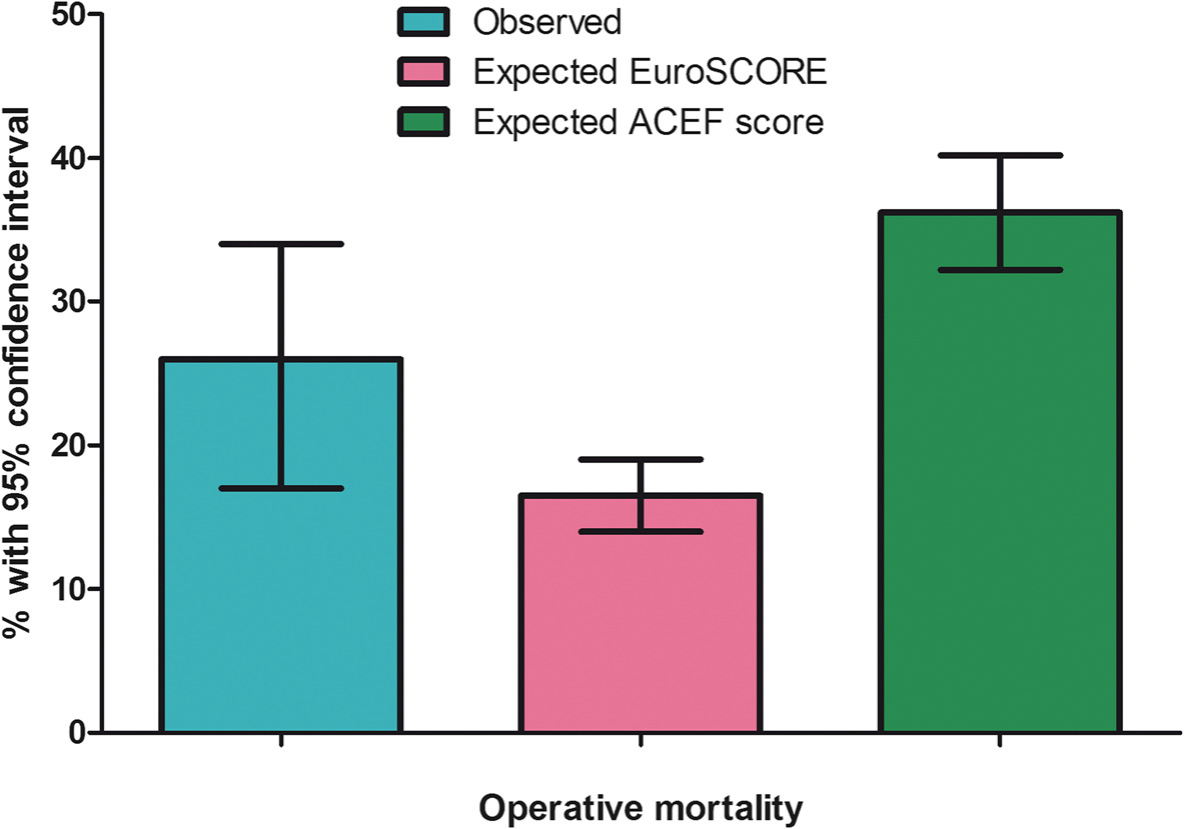
Observed and expected mortality according to the EuroSCORE II and the ACEF score

The observed mortality in our patient population was 26 %. This value was significantly (*P* < 0.05) underestimated by the EuroSCORE II (expected mortality 16.5 %) and significantly (*P* < 0.05) overestimated by the ACEF score (expected mortality 36.2 %), see Fig. 1.

At the diagnostics statistics for prediction capacity, the EuroSCORE II demonstrated a good discrimination ability (c-statistics 0.75) with a chi-square value of 7.9 (*P* = 0.44) at the Hosmer-Lemeshow calibration tests, whereas the ACEF score had no discrimination ability (c-statistics 0.52) and a chi-square value of 9.8 (*P* = 0.20) at the Hosmer-Lemeshow calibration test.

### Multivariable model for second-stage risk stratification

The variables being significantly associated with operative mortality were entered into a multivariable logistic regression analysis. Given the high level of statistical significance, the serum creatinine level was maintained as continuous variable. The preoperative HCT value was analyzed for association with operative mortality using the co-ordinates of a ROC analysis, in order to define an optimal value for dichotomization. This was found at a level of HCT < 34 % (sensitivity 67 %, specificity 67 %) that was defined as preoperative anemia. Therefore, both the presence of PH and anemia were entered into the multivariable model that is shown in Table 2. In this model, serum creatinine levels, the presence of anemia and PH, and surgery under emergency conditions remained independently associated with the operative mortality risk, whereas isolated valve surgery did not.

**Table 2:** Multivariable logistic regression model for operative risk mortality in patients with a risk > 25 % at the first stage

Factor	Regression coefficient	Odds ratio	95 % C.I.	*P* value
Pulmonary hypertension (>55 mmHg)	1.40	4.1	1.1 – 15.2	0.037
Anemia (HCT < 34 %)	1.31	3.7	1.3 – 10.8	0.017
Serum creatinine (mg/dL)	0.98	2.7	1.2 – 6.1	0.020
Emergency surgery	2.90	18.1	2.9 – 114	0.002
Constant	- 3.73			

*C.I*. confidence interval, *HCT* hematocrit

This new model had a good discrimination (c-statistics 0.82), and a chi-square of 4.1 (*P* = 0.77) at the Hosmer-Lemeshow calibration test. The expected mortality rate, at this second-stage risk stratification, was 26.1 % (95 % confidence interval 21.5 % - 30.6 %), in agreement with the observed mortality rate of 26 %.

**Fig. 2:**
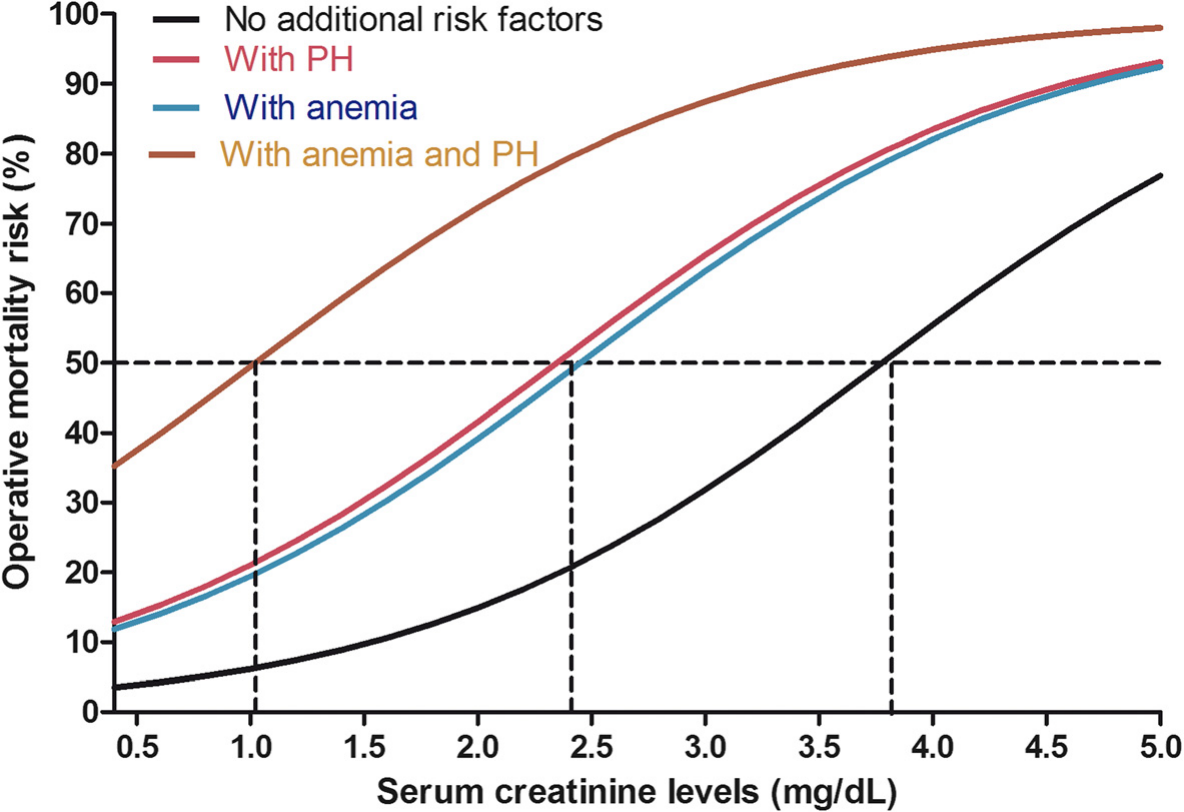
Operative mortality risk as obtained with the double-stage approach, according to serum creatinine levels and the presence of anemia and pulmonary hypertension (PH), in the patients not in emergency conditions

The mortality risk obtained with this two-stage risk stratification model is graphically shown in Fig. 2, for a patient not in emergency conditions, and according to the presence of the different risk factors. A mortality risk of 50 % is reached at a serum creatinine level of 3.8 mg/dL in absence of other risk factors; at 2.4 mg/dL in presence of either anemia or PH; and at a level around 1.0 mg/dL in presence of both anemia and PH.

The exact level of predicted mortality risk can be calculated according to the logistic equation:

$$ \mathrm{Mortality}\ \mathrm{risk}\ \left(\%\right) = 100\ *\frac{e^{\left(-3.73 + \mathrm{creatinine}\ *\ 0.98+\mathrm{pulmonary}\ \mathrm{hypertension}*1.4 + \mathrm{anemia}*1.3\right)}}{\left(1+{e}^{\left[-3.73 + \mathrm{creatinine}*\ 0.98+\mathrm{pulmonary}\ \mathrm{hypertension}*1.4+\mathrm{anemia}*1.3\right]}\right)} $$

where creatinine is expressed in mg/dL, pulmonary hypertension and anemia are binary coded as 0 if absent and 1 if present.

For practical purposes, this equation has been solved (with approximation) for the different combination of factors, as reported in Table 3.

**Table 3:** Predicted operative mortality according to the two-stage model. Stage 2, to be applied in patients with a mortality risk > 25% according to the EuroSCOREII and/or the ACEF Score (stage 1)

Serum creatinine (mg/dL)	Mortality risk (%)		
	Without anemia and PH	With anemia or PH	With both anemia and PH
1.0 or less	5 %	20 %	50 %
1.5	10 %	30 %	60 %
2.0	15 %	40 %	70 %
2.5	20 %	50 %	80 %
3.0	30 %	65 %	90 %
3.5	40 %	75 %	92 %
4.0 or more	50 %	80 %	95 %

PH: pulmonary hypertension (systolic pulmonary pressure > 55 mmHg); anemia: hematocrit < 34%

A reclassification table was applied to the patient population (overall and non-emergency cases). For the purposes of this analysis, we considered the classification into the category of extremely high mortality risk (>50 %) (Table 4). In the overall population, at the first and second stage of risk stratification there was the same number of patients in the extremely high mortality risk class (20 patients), but 26 (25 %) patients were re-classified (12 in the lower class and 14 in the higher class). The observed mortality rate in the extremely high-risk class was 35 % at the first and 70 % at the second-stage classification.

**Table 4:** Re-classification table (from stage 1 to stage 2) for patients with an expected mortality rate > 50 %

	Overall patient population (*N* = 105)			
Risk stratification stage	Number of patients (%)	Number of non-survivors (%)	Number re-classified (higher risk class) (%)	Number re-classified (lower risk class) (%)
First stage (EuroSCORE II or ACEF score)	20 (19)	7 (35)	14 (13)	12 (11)
Second stage	20 (19)	14 (70)		
	Non-emergency patients (*N* = 98)			
First stage (EuroSCORE II or ACEF score)	19 (19)	7 (37)	13 (13)	7 (7)
Second stage	13 (13)	9 (69)		

When applied only to non-emergency cases (98 patients), the reclassification table attributed 19 patients to the extremely high-risk class at the first stage, and 13 at the second stage. Twenty (20.4 %) patients were re-classified, 13 in the lower class and 7 in the higher class. The observed mortality rate in the extremely high-risk class was 42 at the first stage and 69 % at the second-stage classification.

## Discussion

Our study provides confirmation on the well established limitations of the risk stratification scores in high-risk cardiac surgery patients. The novel finding of our study is that the adoption of a double-stage risk stratification model might improve the performance of two of the existing scores.

### Risk stratification scores and high-risk patients in cardiac surgery

The segment of cardiac surgery patients being at very high-risk for operative mortality is a clinical environment where a correct risk stratification is of paramount importance. Much more than in lower risk classes, patients at very high mortality risk pose to the surgeon a difficult challenge, with different therapeutic options, from conventional surgery to transcatheter procedures, even including the possibility of excluding any invasive procedure and limiting the treatment to pharmacological options.

In a patient population with an operative mortality risk > 25 % (based on two validated risk scores), our study confirms that the existing risk models actually offer little information and guidance to the clinicians, leaving them to their clinical judgment in this difficult scenario. The observed mortality rate was actually 28 % less than the expected according to the ACEF score, and more than 50 % higher than expected according to the EuroSCORE II. This last figure is in agreement to what reported by other authors [5–8] who highlighted that in different populations of patients at high surgical risk, the actual mortality exceeded the EuroSCORE II expected mortality by 25 % to 100 %. With respect to the ACEF score, our finding is different from what previously observed in a large validation study, where in high-risk patients the ACEF score was underestimating the mortality risk [11].

There are many possible interpretation for the impressive underestimation of the mortality risk offered by the EuroSCORE II in very high-risk patients. In general, every risk model based on logistic regression analyses tends to reduce its calibration at the two extremes of the risk stratification. This is usually due to the limited number of patients enrolled in the development series being at very low or very high mortality risk, and particularly applies to the very high risk (>25 %) patients. Actually, these represented only 3 % of our patient population. From the original EuroSCORE II article [2] it is difficult to extract the rate of patients being at very high risk in the development series, but the overall patient population is certainly far different from our series, being (as a mean) 10 years younger, having 25 % lower serum creatinine levels, and a lower rate of comorbidities. Mean left ventricular ejection fraction is not reported in the original EuroSCORE II article, but it was certainly much higher than the very low value (median 26 %) reported in our patient population. The group of patients we focused on certainly represents a minority, whose characteristics and risk factors are probably not correctly captured by the EuroSCORE II nor in other existing risk models. Finally, it should be recognized that the concept of mortality utilized in the EuroSCORE II refers to “hospital mortality”, whereas in our series it is “in hospital or 30-days after surgery in discharged patients”. There is a difference between these two values, correctly underlined by the authors of the EuroSCORE II [2], who could assess that 30-days mortality is about 15 % higher than hospital mortality. Even considering this adjustment, the underestimation of the EuroSCORE II in our as in other series of high-risk patients remains important and should be considered an established concept. With respect to the ACEF score, one of the determinants of the mortality risk assessment is the ejection fraction. Our patient population had a very low ejection fraction (12 patients < 20 % ejection fraction) and this resulted in a high ACEF score. It is possible that in a subgroup of patients having such a low ejection fraction, the ACEF score may actually overestimate the mortality risk; additionally, the same methodological considerations apply to the ACEF score as well as to the EuroSCORE II.

### The two-stage risk stratification approach

To overcome this problem in a minority of patient who however deserve a specific tool for risk stratification, our study applied a two-stage approach, based on conventional risk scores (first stage) and further new risk stratification (second stage) using pre-assessed or totally new risk factors. Some of the independent risk factors found in the second-stage model are already present in the ACEF score (serum creatinine) and in the EuroSCORE II (emergency surgery, serum creatinine as a determinant of creatinine clearance, and PH). However, being applied sequentially to the preliminary first-step screening, no intercorrelation concerns can be raised.

One more factor (preoperative anemia) is not included either in the EuroSCORE II or the ACEF score. However, a number of recent studies have highlighted the important role of this factor as an independent determinant of operative mortality [12–15]. Severe anemia (HCT < 30 %) carries an operative mortality risk that is 70 % higher than in patients without this risk factor [12], and even patients with a moderate degree of anemia (HCT 30 %–36 %) experience a significant higher operative mortality [12]. Other studies focused on coronary surgery patients found similar results [13, 14] and a recent study confirmed this finding in a large patient population undergoing different types of cardiac operations [15]. Of notice, in our setting of very high-risk patients, preoperative anemia seems to play an even greater role in determining operative mortality. In absence of other risk factors (no PH and serum creatinine 1.0 mg/dL), the presence of anemia results in a four-times higher mortality risk, and the difference remains more than double at any serum creatinine level between 1.0 and 3.0 mg/dL. For higher serum creatinine levels, the renal risk seems to become dominant, and anemia increases the mortality risk by 70 % if the serum creatinine level is 4.0 mg/dL.

Emergency surgery is a confirmed important factor determining operative mortality even within the setting of very high-risk patients. However, in presence of emergency conditions, there is little room available for risk stratification and decision-making. Therefore, we have focused our analysis on elective and urgent procedures.

It is not within the purposes of the present study to go into the details of the mechanisms linking PH, anemia, and renal function to operative mortality in very high-risk patients undergoing cardiac surgery. However, it should be mentioned that these three factors represent markers of a general severity of the disease and of the heart function. PH is present in a number of severe conditions summarizing the pattern of both systolic and diastolic heart failure, with specific relationship to left-heart valve diseases. Anemia is commonly found within the context of chronic heart failure; additionally, anemia, heart failure and renal dysfunction are the markers of the cardio-renal-anemia syndrome.

### Clinical perspective

Our findings highlight that in a very small but very important segment of cardiac surgery patients being at the top of the operative mortality risk, scores that have been built in the general patient population are probably inadequate. However, it is probably outside the limits of medical statistics to collect the thousands of patients at very high risk that would be necessary to design a dedicated risk score. Our approach is a provocative attempt to overcome this problem using a double-stage risk stratification strategy.

An interesting point in our analysis pertains the age of our patients that was on average 74 years. Elderly patients are more and more referred to cardiac surgery and will increase dramatically in the next years as a result of the increased life expectancy. Even if age was not a significant risk factor at the second stage of risk stratification, a two-stage risk approach may be of particular value in elderly patients as it highlights a number of risk factors that are particularly common in the elderly patient. Within the risk factors increasing the operative mortality risk in very high-risk cardiac surgery patients, a specific mention is deserved by preoperative anemia. Even if both PH and renal dysfunction could theoretically be improved before surgery, anemia remains certainly the most modifiable risk factor [16]. Our data strengthen the need for the implementation of management strategies reduce pre-operative risk in high-risk population. Different strategies can be applied, including iron supplementation and erythropoietin administration. Ferric carboxymaltose provides a considerable increase in hemoglobin values within a reasonable period of time and could be considered when planning cardiac surgery in very high-risk patients. However, adequate trials demonstrating the effectiveness of these strategies in reducing the operative mortality risk in anemic patients undergoing cardiac surgery are presently lacking.

The present study has a number of limitations. The first is its retrospective nature; the second, the (relatively) low number of patients at very high-risk included; the third, the single-center nature of the study. All these limitations do not allow generalizability of our results.

## Conclusion

The present study does not intend to propose a new scoring system. The existing models are probably exhaustive enough for the great majority of the cardiac surgery patient population. However, they are inadequate in those patients (probably no more than 3 % of the general patient population) where the operative mortality risk is so high as to introduce the need for the decision to proceed with surgery or to follow alternative strategies. Our data stress the role of renal function, PH, and anemia in determining the actual outcome of these patients, and provide the surgical team with additional information to be included in the decision-making process. Additionally, our data generate the hypothesis that some of these patients (basically, those with preoperative anemia) may benefit from a careful “re-training” before surgery, based on an improvement of the “modifiable” risk factors. Of course, our data need to be verified in larger patient population and in different institutions, and further studies in this area are highly suggested.

## Abbreviations

ACEF: Age, Creatinine, Ejection Fraction
AKI: Acute Kidney Injury
AUC: Area Under the Curve
EACTS: European Association for Cardio-Thoracic Surgery
ESC: Eurpean Society of Cardiology
EuroSCORE: European System for Cardiac Operative Risk Evaluation
HCT: Hematocrit
IABP: Intra-Aortic Balloon Pump
PH: Pulmonary Hypertension
ROC: Receiver Operating Characteristics
SD: Standard Deviation
STS: Society of Thoracic Surgeons
